# Assessing the causal relationship between psychiatric disorders and obstructive sleep apnea: a bidirectional Mendelian randomization

**DOI:** 10.3389/fpsyt.2024.1351216

**Published:** 2024-02-14

**Authors:** Chuanhao Mi, Ajiao Hou, Yinqin Liu, Xianghua Qi, Jing Teng

**Affiliations:** ^1^ First Clinical Medical College, Shandong University of Traditional Chinese Medicine, Jinan, Shandong, China; ^2^ Key Laboratory of Basic and Application Research of Beiyao, Heilongjiang University of Chinese Medicine, Harbin, Heilongjiang, China; ^3^ Department of Neurology, Affiliated Hospital of Shandong University of Traditional Chinese Medicine, Jinan, Shandong, China

**Keywords:** genome-wide association study, obstructive sleep apnea, causal relationship, psychiatric disorders, mendelian randomization

## Abstract

**Background:**

Extensive observational evidence suggests an association between psychiatric disorders (PDs) and obstructive sleep apnea (OSA), but their causal relationship remains unexplored. The objective of this study was to examine the causal relationship between PDs and OSA.

**Methods:**

Mendelian randomization (MR) analysis was conducted with summary genetic data from the FinnGen and Psychiatric Genomics Consortium (PGC). Inverse-variance weighted (IVW), MR-Egger, weighted median, and weighted mode methods were employed to ascertain causal influence. Sensitivity analysis employing various methodologies assessed the robustness of the findings. Furthermore, multivariable Mendelian randomization (MVMR) was used to clarify if the exposures independently caused OSA.

**Results:**

MR analysis showed that genetically determined major depressive disorder (MDD) increased the risk of OSA (IVW odds ratio [OR]: 1.377, 95% confidence interval [CI]: 1.242–1.526, *P* = 1.05×10^-9^). Sensitivity analysis showed no evidence of pleiotropy and heterogeneity. In MVMR, the significant association persisted after adjusting for BMI, smoking, and alcohol consumption. No conclusive evidence indicated the causal impact of other psychological characteristics on OSA. In the reverse MR analyses, there was no causal effect of OSA on PDs.

**Conclusion:**

This study suggests a causal effect of MDD on OSA risk. Further research is needed to confirm these findings and understand how MDD contributes to OSA development, potentially aiding in reducing OSA incidence.

## 1 Introduction

Obstructive sleep apnea (OSA) is a prevalent chronic sleep disorder characterized by recurring episodes of partial or complete collapse of the upper airway during sleep. This leads to intermittent hypoxia, sympathetic overactivity, sleep fragmentation, and disruption of physiological homeostasis (1). The prevalence of OSA varies, affecting 9% to 38% of the general population, with higher rates observed in men (13%-33%) compared to women (6-19%) (2). Numerous studies have established a link between OSA and an increased risk of several metabolic and cardiovascular conditions, including coronary heart disease, type 2 diabetes mellitus, hypertension, and stroke (3). Therefore, understanding OSA’s pathophysiology and potential risk factors is critical for developing novel prevention and intervention strategies.

Psychiatric disorders (PDs), encompassing a range of complex cognitive psychological syndromes, have emerged as a major public health concern with significant morbidity and mortality rates. These disorders are estimated to affect about 22.1% of the global population (4). The potential link between PDs and OSA has recently garnered increasing research interest (5–7). Numerous studies indicate a high prevalence of OSA in patients with psychiatric conditions (5, 8, 9), with elevated rates of OSA observed explicitly in patients with post-traumatic stress disorder (PTSD) and bipolar disorder (BIP) compared to the general population (10–12). Conversely, increased incidences of PDs are reported in individuals with OSA (13, 14), with depression and anxiety disorder (ANX) being more common in OSA patients than in non-OSA counterparts (15, 16). Moreover, several studies suggest that treatment of OSA with continuous positive airway pressure (CPAP) can lead to improvements in both OSA symptoms and psychiatric comorbidities like depression and PTSD (17–19). However, accurately diagnosing PDs in OSA patients is challenging due to the overlap of symptoms such as excessive daytime sleepiness, cognitive impairment, and mood changes (7). Additionally, factors like alcohol consumption, smoking, and body mass index (BMI) can confound the relationship between OSA and PDs (20, 21), leading to potential biases in observational studies. Therefore, the causality between OSA and PDs remains ambiguous and requires further exploration.

MR is an epidemiological method that employs genetic variants strongly associated with exposure as instrumental variables (IVs) to elucidate causal relationships between risk factors and outcomes (22). Due to the random inheritance of genetic variations and their stability post-pregnancy, MR minimizes the influence of external factors on causality, significantly reducing potential biases and reverse causation. This strengthens the robustness of causal inferences, addressing limitations commonly associated with observational studies (23). Widely applied to validate findings from observational research (24), MR was employed in this study to conduct a two-sample bidirectional analysis. This analysis aimed to investigate the causal associations between five PDs—MDD, PTSD, BIP, ANX, and schizophrenia (SCZ)—and OSA.

## 2 Materials and methods

### 2.1 Study design

**Figure 1:**
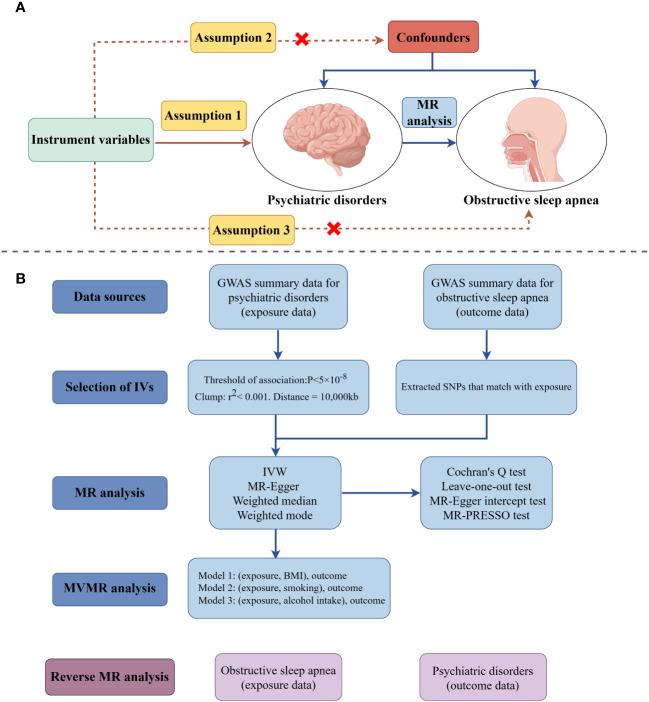
The schematic illustrates the causal relationship between psychiatric disorders and obstructive sleep apnea through MR analyses (drawing by Figdraw). **(A)** Principles of Mendelian Randomization; **(B)** The flowchart of the MR analysis. (MR, Mendelian randomization; MVMR, multivariable Mendelian randomization; SNPs, single- nucleotide polymorphisms; IVs, instrument variables; IVW, inverse-variance weighted; BMI, body mass index; GWAS, genome-wide association study).

A two-sample univariable bidirectional MR analysis was initially performed to investigate the causal effects between PDs and OSA. Recognizing that BMI, smoking, and alcohol consumption are significant risk factors for OSA development, multivariable MR (MVMR) analyses were subsequently conducted for outcomes causally linked to OSA. These analyses aimed to ascertain whether the exposures have independent causal effects on OSA. To ensure the reliability of MR results, three critical assumptions must be met: (i) the IVs must be strongly correlated with the exposures, (ii) the IVs should not be significantly associated with confounding factors, and (iii) the IVs must influence the outcomes solely through the exposures (25). The research design is depicted in **Figure 1**, created using Figdraw (www.figdraw.com).

### 2.2 Data source

Genome-wide association studies (GWAS) aim to identify relationships between differences in DNA sequences and specific traits by examining individuals with diverse phenotypes and analyzing their genotypes at several single-nucleotide polymorphisms (SNPs) locations (26). As data-sharing has advanced, GWAS has discovered several significant variants related to PDs and OSA. These variations provide valuable IVs for conducting a robust MR analysis to investigate the causal relationship between PDs and OSA. To mitigate the risk of pleiotropic bias in cross-lineage cases, this study incorporated publicly available GWAS results of five PDs, and OSA conducted within a European population.

The most recent GWAS data for OSA was obtained from the FinnGen (27), which included a total of 38,998 patients and 336,659 controls. The patients are identified using the Finnish National Hospital Discharge Registry and the Causes of Death Registry. OSA was diagnosed depending on the International Statistical Classification of Diseases and Related Health Problems, 9th edition (ICD-9: 3472A) and 10th edition (ICD-10: G47.3), which are based on subjective symptoms, clinical examination, and sleep registration, utilizing the apnea-hypopnea index of five per hour or the respiratory event index of five per hour. All association tests for all sources were adjusted for principal variables, including age and sex. The Psychiatric Genomics Consortium (PGC) provided summarized GWAS data on the five PDs:

The GWAS data for MDD was obtained from Howard DM et al.’s GWAS meta-analysis (28). It consists of PGC, UK Biobank, and 23andMe, three large-scale GWAS. We included the complete summary statistics from 2 cohorts to conduct bi-directional MR analysis because, among these three GWAS, the summary statistics for all examined genetic variants were only publically available for UK Biobank and PGC (which contains 170,756 cases and 344,901 controls). Howard DM et al. employed the inclusive criteria for MDD within UK Biobank, which was determined based on participants’ answers to the questions ‘Have you ever sought medical help from a general practitioner or psychiatrist for nerves, anxiety, tension, or depression?’ MDD was diagnosed in PGC cohorts using internationally recognized criteria, such as DSM-IV, ICD-9, or ICD-10.

The GWAS data for ANX were obtained from the GWAS meta-analysis conducted by Otowa T et al. (29), which included 7 distinct studies with a combined sample size of 5580 cases and 11730 controls. The identification of ANX primarily relied on the Diagnostic and Statistical Manual of Mental Disorders (DSM) and utilized standardized evaluation tools (30).

The GWAS data for BIP were obtained from a meta-analysis of 57 cohorts from Europe, North America, and Australia undertaken by Mullins N et al. (31). These cohorts included a total of 41,917 cases and 371,549 controls in European heritage. Cases that satisfied the global consensus criteria (DSM-IV, ICD-9, or ICD-10) for BIP were determined through formal diagnostic interviews, clinician checklists, or medical records reviews.

The largest GWAS meta-analysis, carried out by Trubetskoy V et al. (32), yielded the GWAS data for SCZ. The SCZ samples were from several SCZ studies. The DSM-IV or ICD-10 diagnostic criteria were used to make the diagnoses. For the purpose of performing a bi-directional MR study, we only included the SCZ meta-analysis in participants of European ancestry. This analysis included 53,386 cases and 77,258 controls.

The GWAS data for PTSD were obtained from the GWAS meta-analysis of an ancestrally diverse group from 60 different PTSD studies carried out by Nievergelt CM et al. (33), ranging from large cohorts with self-reported PTSD symptoms to clinically deeply characterized small patient groups. The diagnosis of PTSD was determined using a variety of instruments and DSM versions (DSM-III-R, DSM-IV, DSM-5). To conduct a bi-directional MR analysis, we exclusively included the PTSD meta-analysis in participants of European ancestry, which included 23,212 cases and 151,447 controls.

Additionally, summary-level data for BMI, smoking, and alcohol intake were acquired from corresponding publicly available GWAS. The Genetic Investigation of Anthropometric Traits (GIANT) Consortium, which includes 681,275 samples with European ancestry, provided the GWAS data for BMI (34). The GWAS data on alcohol consumption and smoking initiation were taken from the GWAS meta-analysis conducted by the Sequencing Consortium of Alcohol and Nicotine Use (GSCAN) and the GWAS, which included up to 1.2 million people with European ancestry (35). The binary phenotype of smoking initiation indicates whether a person has ever smoked regularly throughout their life. The average number of drinks a person reported having each week, regardless of the type of alcohol, was used to characterize alcohol consumption. As all original studies were public, anonymized, and de-identified, ethical approval did not apply to this study. **Table 1** provides further details about the GWAS dataset included in this analysis.

**Table 1:** The GWAS dataset included in this MR study.

Traits	Sample size(cases/controls)	Population	Consortium	PMID	IEU Open GWAS ID
Exposure					
MDD	500,199 (170,756/329,443)	European	PGC	30,718,901	ieu-b-102
ANX	17,310 (5,580/11,730)	European	PGC	26,754,954	–
BIP	413,466 (41,917/371,549)	European	PGC	34,002,096	ieu-b-5110
SCZ	130,644 (53,386/77,258)	European	PGC	35,396,580	ieu-b-5102
PTSD	174,659 (23,212/151,447)	European	PGC	31,594,949	–
Outcome					
OSA	375,657 (38,998/336,659)	European	FinnGen	G6_SLEEPAPNO	–
Confounders					
BMI	681,275	European	GIANT	–	ieu-b-40
Smoking initiation	607,291 (311,629/321,173)	European	GSCAN	–	ieu-b-4877
Alcoholic drinks per week	335,394	European	GSCAN	–	ieu-b-73

MDD, major depressive disorder; SCZ, schizophrenia; BIP, bipolar disorder; ANX, anxiety disorder; PTSD, post-traumatic stress disorder; OSA, obstructive sleep apnea; BMI, body mass index; PGC, Psychiatric Genomics Consortium; GIANT, Genetic Investigation of Anthropometric Traits; GSCAN, Sequencing Consortium of Alcohol and Nicotine Use; PMID, PubMed Unique Identifier; GWAS, genome-wide association study; IEU, Integrative Epidemiology Unit.

### 2.3 Instrument variables selection

A specific methodology was employed to select IVs to adhere to the three core assumptions of MR analysis. Genome-wide significant SNPs (P<5×10^−8^) were initially extracted from exposure GWAS. For the GWAS of ANX and PTSD, suggestively significant SNPs (*P <*5×10^−6^) were also used as IVs to ensure comprehensive results. Subsequently, linkage disequilibrium (LD) clumping (*R^2^* < 0.001, clumping distance = 10,000 kb), based on the European 1,000 Genomes Project reference panel, was performed to select independent significant SNPs. The harmonization process involved removing palindrome SNPs with intermediate or incompatible allele frequencies. The statistical strength of each SNP was then evaluated by calculating the F-statistic (beta^2^/se^2^) (36, 37), with an F-statistic > 10 indicating the absence of bias due to weak IVs. Additionally, MR pleiotropy residual sum and outlier (MR-PRESSO) test analysis were conducted to eliminate outlier IVs. A stringent filtering step was adopted, discarding SNPs with a *P*-value < 1 in the outlier test and repeating this process until no outliers remained (38). Finally, the Steiger test was applied to each SNP to ascertain if the *R^2^* of the exposure exceeded that of the outcome, excluding SNPs where the test indicated a ‘FALSE’ direction.

### 2.4 Univariable MR analyses

This study used the inverse-variance weighted (IVW) as the primary analytical approach to assess the causal relationship between PDs and OSA. This method aggregates Wald ratio estimates for each instrumental SNP using a meta-analysis-like model to derive overall effect estimates (39). Given that IVW yields unbiased causal estimates only when all IVs are valid, additional methods were employed to ensure accuracy and stability in cases of assumption violations. These methods included weighted mode, weighted median, and MR-Egger. The weighted median method can offer a consistent estimate even if up to 50% of the IVs are invalid, albeit with reduced statistical power (40). Under the Instrument Strength Independent of Direct Effect (InSIDE) assumption, MR-Egger regression allows all genetic variants to be potentially invalid IVs. It provides an intercept term for estimating average pleiotropic effects across genetic variants (41). Despite stringent control measures, the MR-PRESSO method might still detect horizontal pleiotropy in the global test, potentially due to InSIDE assumption violations (42). Therefore, if the MR-PRESSO international test detected pleiotropy or if the MR-Egger intercept’s *P*-value was below 0.05, results from the weighted median method were prioritized. The weighted mode approach is applicable even when most IVs do not fulfill the causal inference prerequisites in MR as long as most IVs yield consistent causal estimations (41).

### 2.5 Sensitivity analysis

A series of essential sensitivity assessments were conducted to evaluate the results’ reliability and stability. The Cochran Q statistic (MR-IVW) was employed to assess heterogeneity among the effect sizes derived from the selected genetic IVs (43). A *P*-value greater than 0.05 was indicative of negligible heterogeneity. The MR-PRESSO method and MR-Egger intercept were then applied to detect horizontal pleiotropy (42). Furthermore, a leave-one-out analysis addressed potential biases due to horizontal pleiotropy arising from any SNP. For multiple testing adjustments, a Bonferroni-corrected *P*-value threshold was set at 0.05/5, while a *P* < 0.05 was considered nominally significant. Additionally, reverse MR analysis was conducted to explore the possibility of reverse causality, precisely the effect of OSA on PDs.

### 2.6 Multivariable MR analyses

Two distinct methodologies were implemented to mitigate the influence of potential confounders. The first step involved the identification and exclusion of pleiotropic SNPs associated with known confounders like BMI, smoking, and alcohol consumption. This identification was conducted using the PhenoScanner V2 database (44). After removing SNPs linked to these confounding variables, a reanalysis was carried out using the remaining IVs. Additionally, MVMR analysis was used. An improved version of MR named MVMR enables the simultaneous analysis of several genetic variations linked to various exposures (45). This method is advantageous in discerning whether the exposures under study have independent effects on the outcome, in this case, OSA. In the second approach, MVMR analysis was explicitly used to determine if these exposures had independent causal effects on OSA. These analyses were conducted using various packages in R Software 4.3.0, including ‘TwoSampleMR’ (version 0.5.7), ‘MendelianRandomization’ (version 0.8.0), and ‘MRPRESSO’ (version 1.0).

## 3 Results

### 3.1 Selection of IVs

A thorough quality control process led to the selection of 41, 6, 38, 137, and 20 SNPs as IVs for MDD, ANX, BIP, SCZ, and PTSD, respectively, to evaluate their causal effect on OSA. Conversely, in the reverse MR analysis, 11, 14, 14, 16, and 18 SNPs were chosen as IVs for OSA to assess its causal impact on the five PDs. The F-statistic for each selected SNP was greater than 10, indicating the absence of weak instrument bias. Detailed information about the IVs ultimately included in our MR analysis can be found in **Supplementary Tables S1–S10**.

### 3.2 Causal effect of PDs on OSA

The univariable MR analysis revealed that genetically predicted MDD was associated with an increased risk of OSA (IVW OR: 1.377, 95% CI: 1.242–1.526, *P* = 1.05×10^-9^). This association was corroborated by the weighted median method (OR: 1.301, 95% CI: 1.135–1.491, *P* = 1.576×10^-4^). Although MR-Egger and weighted mode methods provided consistent estimates, they were not statistically significant. No heterogeneity was observed in Cochran’s Q test (*P* = 0.084), and both the MR-Egger intercept (*P* = 0.480) and MR-PRESSO test (*P* = 0.114) indicated an absence of significant directional pleiotropy for the IVs. Leave-one-out analyses further confirmed that individual SNPs did not influence the causal relationship. For SCZ, despite stringent controls and no outliers detected pre-MR analysis, the MR-PRESSO global test indicated pleiotropy among IVs (*P* = 0.037), suggesting unreliability of causation estimates from IVW and MR-Egger due to violation of the InSIDE assumption (38). However, estimates from the weighted median method were also non-significant (OR: 0.997, 95% CI: 0.964–1.032, *P* = 0.884). MR analysis indicated no causal effect of genetically predicted ANX, BIP, and PTSD on OSA. Cochran’s Q test detected no heterogeneities except for PTSD (*P* = 0.042), and no notable directional pleiotropies were observed in the MR-Egger intercept and MR-PRESSO test. Detailed results of the causal effects of PDs on OSA, including outcomes of the four causal estimation methods, pleiotropy tests, and heterogeneity tests, are presented in **Table 2**. The scatter, funnel, and forest plots are shown in **Supplementary Figure S1**.

**Table 2:** The causal effect of psychiatric disorders on OSA.

Exposure	Method	nSNPs	OR	95% CI	*P*-value	Heterogeneity	Pleiotropy	
						Q_*P*-value	Egger intercept *P*-value	MR-PRESSO *P*-value
MDD	IVW	41	1.377	1.242-1.526	1.05×10^-9^	0.084		0.114
	MR-Egger	41	1.104	0.597-2.041	0.754	0.078	0.480	
	Weighted median	41	1.301	1.135-1.491	1.576×10^-4^			
	Weighted mode	41	1.253	0.911-1.724	0.173			
ANX	IVW	6	1.017	0.979-1.056	0.397	0.482		0.509
	MR-Egger	6	0.977	0.872-1.095	0.709	0.411	0.508	
	Weighted median	6	0.991	0.939-1.045	0.729			
	Weighted mode	6	0.987	0.923-1.057	0.730			
BIP	IVW	38	1.016	0.974-1.063	0.434	0.342		0.343
	MR-Egger	38	0.959	0.732-1.256	0.763	0.308	0.665	
	Weighted median	38	0.992	0.935-1.053	0.780			
	Weighted mode	38	0.987	0.876-1.114	0.838			
SCZ	IVW	137	1.017	0.992-1.043	0.175	0.035		0.037
	MR-Egger	137	0.946	0.852-1.051	0.303	0.041	0.165	
	Weighted median	137	0.997	0.964-1.032	0.884			
	Weighted mode	137	0.926	0.822-1.044	0.211			
PTSD	IVW	20	1.032	0.985-1.081	0.189	0.042		0.052
	MR-Egger	20	1.070	0.980-1.168	0.150	0.044	0.352	
	Weighted median	20	1.031	0.971-1.096	0.320			
	Weighted mode	20	1.057	0.982-1.137	0.159			

MDD, major depressive disorder; SCZ, schizophrenia; BIP, bipolar disorder; ANX, anxiety disorder; PTSD, post-traumatic stress disorder; OSA, obstructive sleep apnea; BMI, body mass index; nSNPs, number of single-nucleotide polymorphisms; IVW, inverse-variance weighted; OR, odds ratio; CI, confidence interval; MR, Mendelian randomization; MR-PRESSO, MR pleiotropy residual sum and outlier.

### 3.3 Causal effect of OSA on PDs

To elucidate the precise causal relationship between PDs and OSA, a reverse MR analysis was conducted, positioning OSA as the exposure and PDs as the outcomes. However, no significant causal effects of OSA were found on five PDs across all MR methods. Additionally, no heterogeneities or directional pleiotropies were observed in the reverse MR analysis. Detailed results of the causal effects of OSA on PDs, encompassing outcomes from the four causal estimation methods, pleiotropy tests, and heterogeneity tests, are provided in **Supplementary Table S11**. The associated scatter and forest plots are illustrated in **Supplementary Figure S2**.

### 3.4 Results of multivariable MR analysis

Employing the online tool PhenoScanner V2, the study identified that rs10235664, rs17641524, rs2568958, rs30266, and rs9831648 correlated with BMI, while rs2232423 and rs4799949 linked to smoking, and rs4936276 and rs66511648 were associated with alcohol consumption. Upon excluding these SNPs from the IVs, the significant causal relationship between MDD and OSA remained intact (OR: 1.373, 95%CI: 1.230–1.532, *P* = 1.60×10^-8^). No considerable heterogeneity or pleiotropy was observed (**Supplementary Table S12**). Subsequently, an MVMR analysis was performed to investigate MDD’s direct effect on OSA, adjusting for BMI, smoking, and alcohol consumption. The MVMR analysis demonstrated that MDD still exerted an immediate impact on OSA. Detailed results of the MVMR analysis are presented in **Table 3**.

**Table 3:** Results of multivariable MR analysis.

Exposure	Outcome	Method	OR	95% CI	*P*-value
Adjusted for BMI	OSA	IVW	1.136	1.005-1.284	0.041
		MR-Egger	1.338	1.106-1.618	2.67×10^-3^
		MR-Lasso	1.167	1.056-1.288	2.33×10^-3^
Adjusted for smoking	OSA	IVW	1.337	1.157-1.544	8.06×10^-5^
		MR-Egger	1.270	1.016-1.586	0.036
		MR-Lasso	1.407	1.263-1.566	4.84×10^-10^
Adjusted for alcohol consumption	OSA	IVW	1.298	1.133-1.487	1.71×10^-4^
		MR-Egger	1.651	1.247-2.185	4.58×10^-4^
		MR-Lasso	1.312	1.189-1.449	6.82×10^-8^

OSA, obstructive sleep apnea; BMI, body mass index; OR, odds ratio; CI, confidence interval; IVW, inverse-variance weighted.

## 4 Discussion

Although there is a strong correlation between PDs and OSA based on observation, the exact cause relationship is yet unknown. In this study, a bidirectional two-sample MR analysis was conducted to investigate the causal effects of five PDs (MDD, ANX, SCZ, BIP, and PTSD) on OSA. The analysis revealed that genetically determined MDD was associated with an increased risk of OSA, a finding that remained consistent and robust across various sensitivity analyses. Further MVMR analysis confirmed that the impact of MDD on OSA persisted even after adjusting for BMI, smoking, and alcohol consumption. Conversely, the inverse MR analysis indicated that genetically predicted OSA did not have a causal effect on any of the five PDs.

The established association of both MDD and OSA with increased cardiovascular morbidity and mortality (46, 47), combined with their frequent comorbidity, has garnered significant attention. Studies indicate that MDD patients often have elevated pro-inflammatory cytokines, potentially causing neural injury and disrupting homeostatic and circadian processes of the sleep-wake cycle. This may increase the risk of OSA and exacerbate its symptoms (48, 49). Patients undergoing evaluation of OSA in a sleep laboratory also frequently use antidepressants; up to 25% of patients use drugs for depression (50). Patients who had been administered antidepressants had a higher chance of having OSA patients (51). There may be a relationship between MDD, OSA and obesity. Research has demonstrated that being fat increases the likelihood of having moderate-to-severe OSA (more than 50% of those with this condition are obese), and it also increases the likelihood of developing MDD and its onset (52). Observational studies reveal a greater incidence of OSA in individuals with MDD. For example, a large cohort study found that patients with depression had a higher incidence of OSA diagnoses (7.4% vs. 2.9%) compared to non-depressed controls (53). A population-based longitudinal study in Taiwan also reported an increased likelihood of OSA diagnosis in patients with depression compared to controls (54). Our findings corroborate these observations, suggesting that genetically determined MDD is associated with an elevated risk of OSA. Because of the potential confounding effects of alcohol consumption, smoking, and BMI on the association between OSA and PDs, MVMR analysis was carried out. The MVMR analysis has verified that MDD continues to have a direct impact on OSA, even after accounting for factors such as BMI, alcohol consumption, and smoking. However, research on the prevalence of OSA in MDD patients is limited, potentially due to patient non-compliance, resulting in underdiagnosis (55). Therefore, while these MR findings are promising, they should be interpreted cautiously. Further research is required to validate these results and understand the mechanisms through which MDD influences OSA development, which could significantly reduce OSA incidence.

In this study, no causal link was found between psychiatric conditions like SCZ, BIP, PTSD, and OSA, diverging from previous research outcomes. Earlier observational studies suggested an increased OSA risk among SCZ patients compared to those without SCZ (56, 57) and a higher OSA prevalence in individuals with PTSD and BIP than in the general population (10–12). However, these contrasts with our MR analysis might stem from confounding elements in observational studies. For instance, the increased OSA prevalence and severity in patients on antipsychotic medications could be a consequence of side effects like extrapyramidal symptoms or metabolic syndrome. These effects can increase upper respiratory tract resistance and disrupt sleep breathing, escalating OSA risks (58, 59). Unraveling the complex relationship between these PDs and OSA demands further population-based and experimental research. A deeper understanding of these links could be pivotal in the early prevention and diagnosis of OSA.

A reverse MR analysis was conducted to determine the causal relationship between PDs and OSA. However, no significant causal effects of OSA were found on PDs in reverse MR. This contrasts with previous studies suggesting that OSA patients exhibit a dysfunctional immune response, characterized by an increase in pro-inflammatory cytokines like C-reactive protein, IL-6, and TNF-α, which could influence the onset and maintenance of MDD (60, 61). A meta-analysis of five longitudinal studies indicated a higher risk of developing depression in individuals with OSA compared to those without (52). Furthermore, a cross-sectional study reported a higher frequency of anxiety in OSA patients than in the general population (62). The discrepancy between these observational studies and our MR findings could be due to several factors. There is a similarity between the symptoms of OSA and PDs (6, 7). The diagnostic criteria for OSA encompass symptoms such as drowsiness, exhaustion, or sleeplessness, which are also shared diagnostic criteria for some PDs (63). For instance, the Diagnostic and Statistical Manual of Mental Disorders, Fourth Edition, Text Revision (DSM-IV-TR) and the International Classification of Sleep Disorders, Second Edition (ICSD-2) diagnostic criteria for MDD and OSA, respectively, show that these two disorders share symptoms such as daytime tiredness, irritability, and poor concentration (64). Sleep laboratories frequently assess the depressed symptoms of their patients through the use of screening questionnaires (65). The scales, such as the Hamilton Depression Scale (HAMD), the Montgomery-Asberg Depression Rating Scale, and the Beck Depression Inventory (BDI), assess several aspects of sleep, including drowsiness, sleep disturbance, sleep quality, insomnia, and exhaustion. Hence, symptoms of OSA can impact the results of widely employed psychological evaluation instruments. When the HAMD questionnaire asks patients to indicate if they have symptoms such as feeling weak or weary and being easily awakened from sleep, the scores on the psychiatric assessment may be inflated if the patient has sleep-related symptoms caused by OSA. In observational studies, the presence of confounding scores on psychiatric exams may result in an exaggerated diagnosis of PDs. Secondly, the frequent comorbidity of OSA with conditions like insomnia, stroke, diabetes, and cardiovascular disease (66, 67) could confound the association between OSA and PDs. For example, one study found that patients with both OSA and insomnia had significantly higher rates of PDs than those with OSA alone (68).

This study presents several key strengths. Firstly, while previous epidemiological studies have indicated a contentious relationship between PDs and OSA, potentially confounded by extraneous factors and reverse causality, our MR analysis, particularly the MVMR approach, mitigates such biases inherent in observational studies. Secondly, the MR analysis leveraged data from large-scale, published GWAS pooled studies, enhancing the robustness and power of our findings. Finally, this MR study explored the potential causal relationships across a broad spectrum of PDs and OSA, addressing research gaps in existing observational studies and significantly expanding the scope of current research in this domain.

However, recognizing the limitations of this investigation is essential. First and foremost, the GWAS primarily consisted of individuals of European ancestry, necessitating a cautious approach to generalizing our findings to other ethnic groups. Furthermore, the severity of OSA can influence the cause-and-effect connection between OSA and PDs. A comprehensive study including a large group of participants has demonstrated that transitioning from one degree of OSA severity to the next is linked to a 1.8-fold rise in the adjusted probability of developing depression (69). However, no subgroup analysis of OSA severity was performed in our study due to a lack of essential data, which limited our investigation into the causal relationship between PDs and various degrees of OSA. When GWAS with severity distribution becomes publicly available, further in-depth MR analysis will be required. Finally, while specific known confounders were identified and adjusted for, it is essential to acknowledge that residual confounding factors might still influence the study results.

## 5 Conclusion

This study provides proof that genetically determined MDD is associated with an increased risk of OSA. However, it is crucial to recognize that these findings are based on statistical analyses and necessitate additional empirical research. Further investigation is necessary to confirm these results and to explore the mechanisms by which MDD contributes to the development of OSA. Such understanding could help develop strategies to reduce the incidence of OSA.

## Data availability statement

The original contributions presented in the study are included in the article/**Supplementary Material**. Further inquiries can be directed to the corresponding author.

## Ethics statement

Our analyses used published studies or publicly available GWAS abstract data. All studies were conducted with the approval of the appropriate institutional ethics committees, and therefore did not require additional ethical approval.

## Author contributions

CM: Conceptualization, Software, Writing – original draft, Writing – review & editing. AH: Writing – original draft. YL: Writing – original draft. XQ: Writing – review & editing. JT: Writing – review & editing.
