# Supplementary material for: Assessing the causal relationship between psychiatric disorders and obstructive sleep apnea: a bidirectional Mendelian randomization
Source: Front Psychiatry. 2024 Feb 14;15:1351216. doi: 10.3389/fpsyt.2024.1351216 (PMC10903261; doi:10.3389/fpsyt.2024.1351216)
Supplement: Supplementary file 1 [file DataSheet_1.docx]

**Supplementary Figure S1**


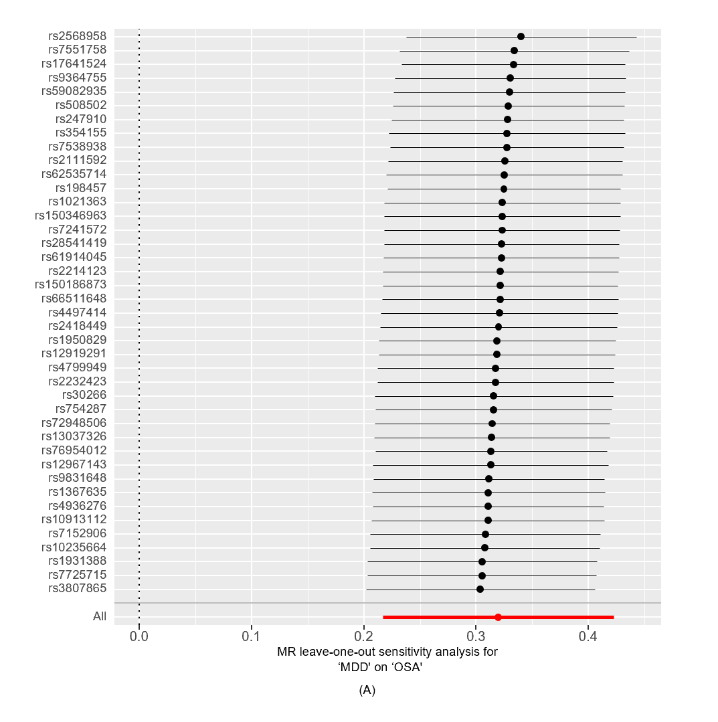

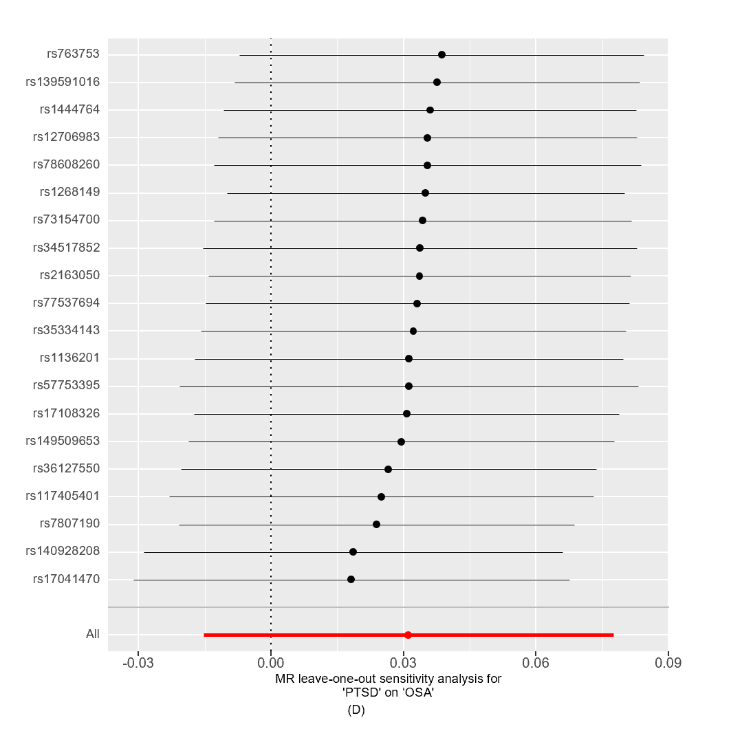

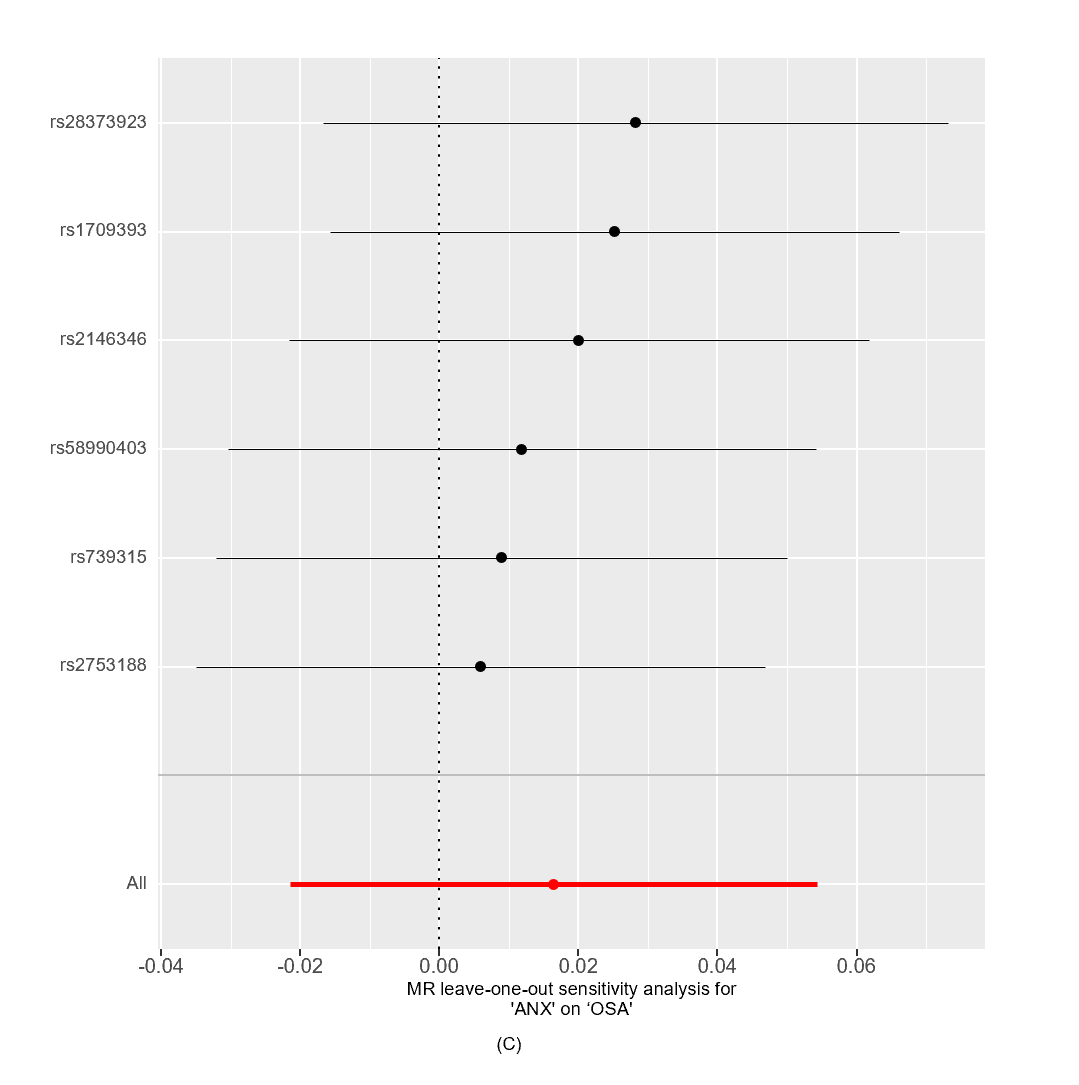

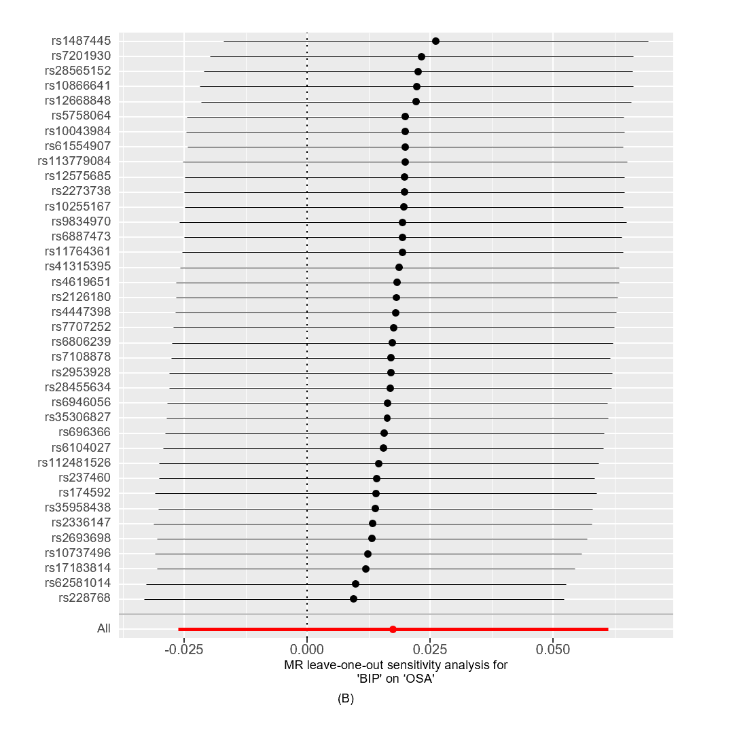
1. Leave-one-out analysis for psychiatric disorders on obstructive sleep apnea.


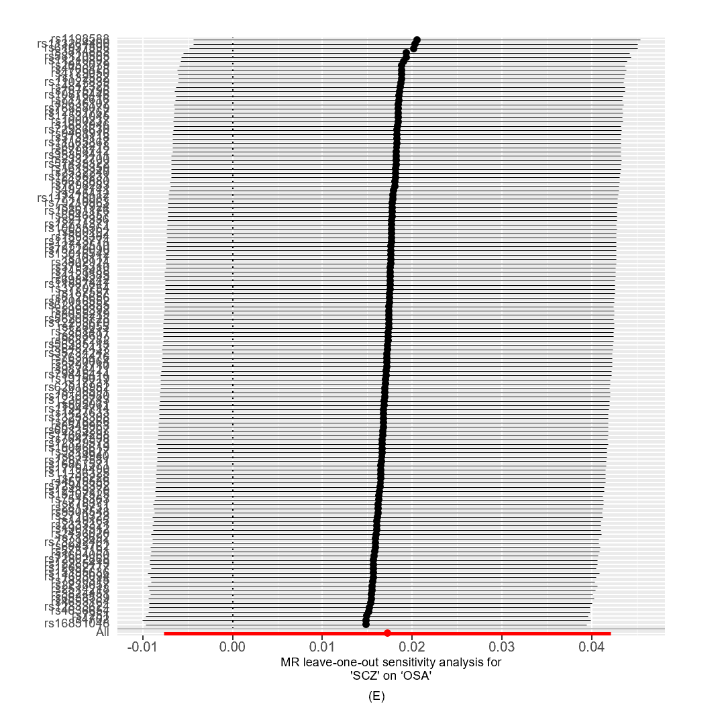


Abbreviations: MDD, major depressive disorder; SCZ, schizophrenia; BIP, bipolar disorder; ANX, anxiety disorder; PTSD, post-traumatic stress disorder; OSA, obstructive sleep apnea.


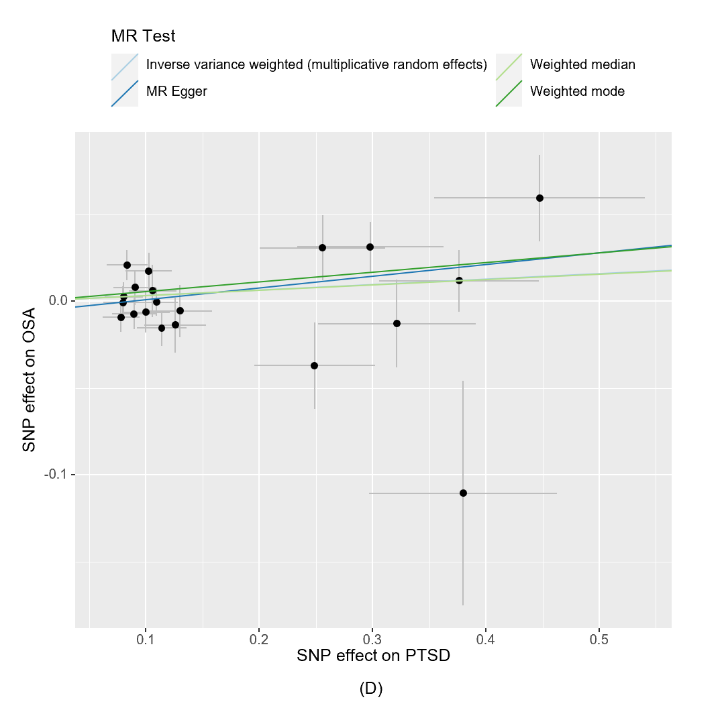

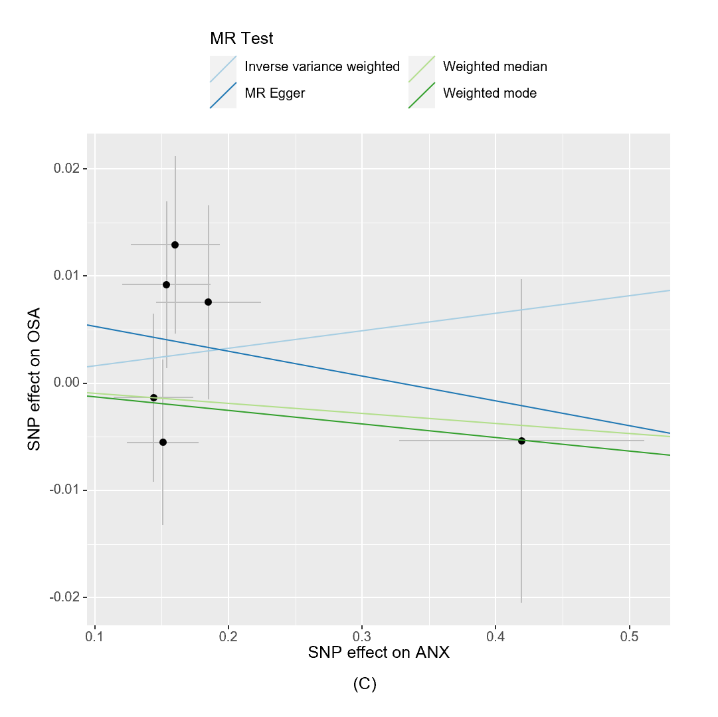

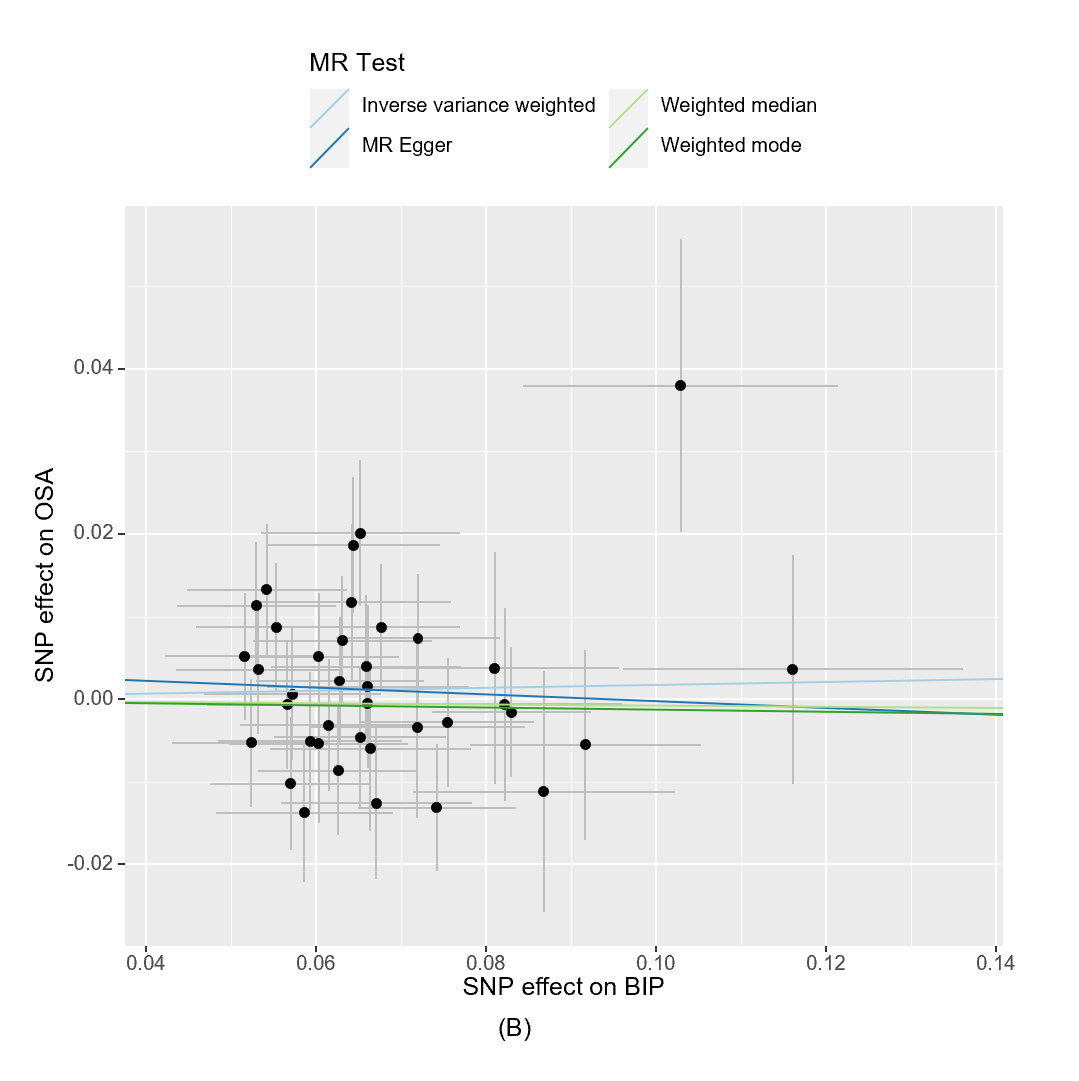
2. Scatter plots for psychiatric disorders on obstructive sleep apnea.


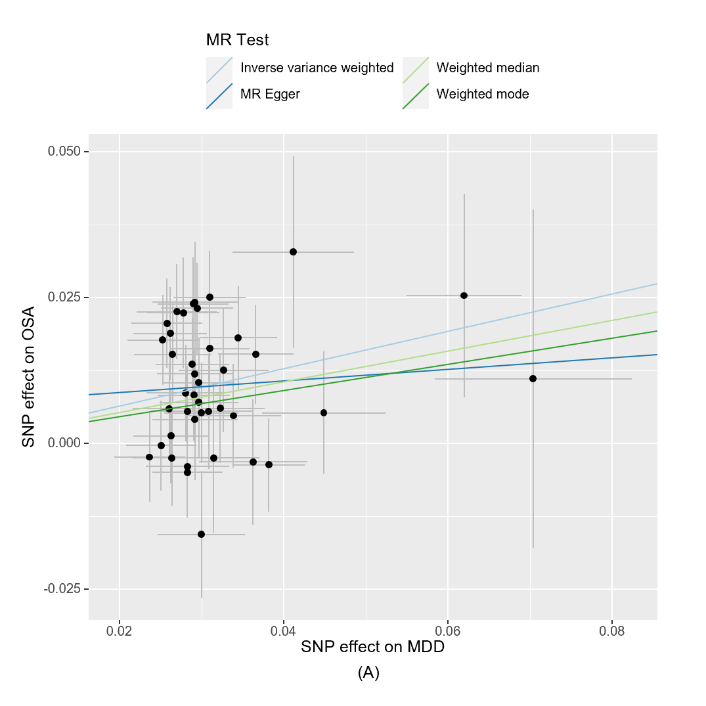


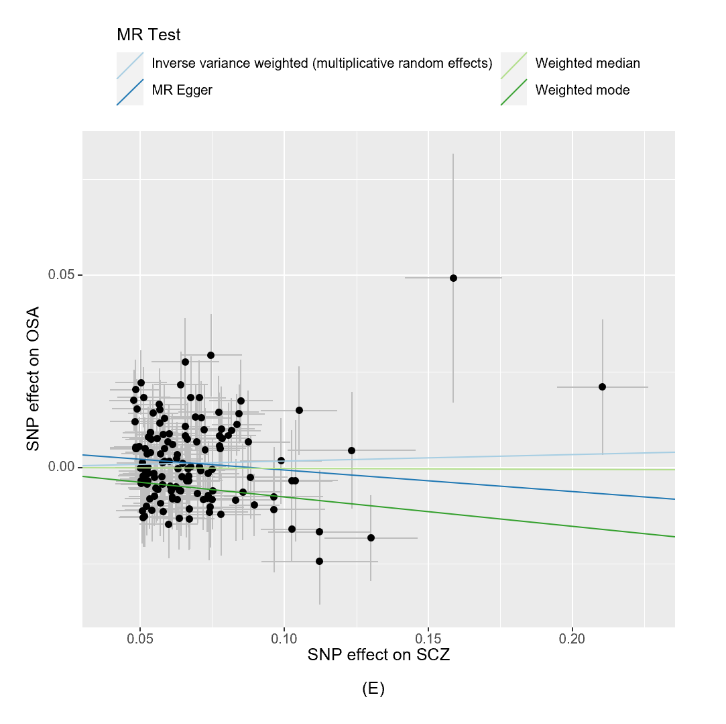


Abbreviations: MDD, major depressive disorder; SCZ, schizophrenia; BIP, bipolar disorder; ANX, anxiety disorder; PTSD, post-traumatic stress disorder; OSA, obstructive sleep apnea.


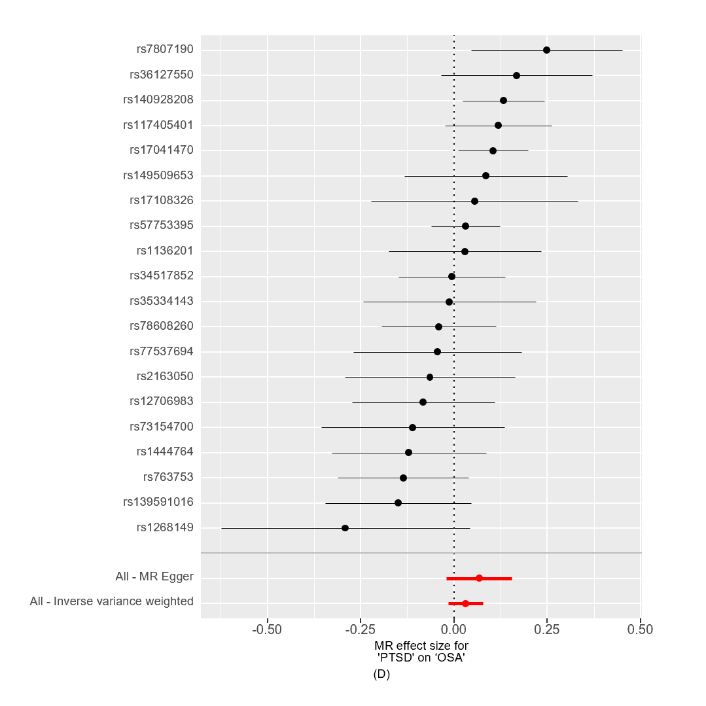

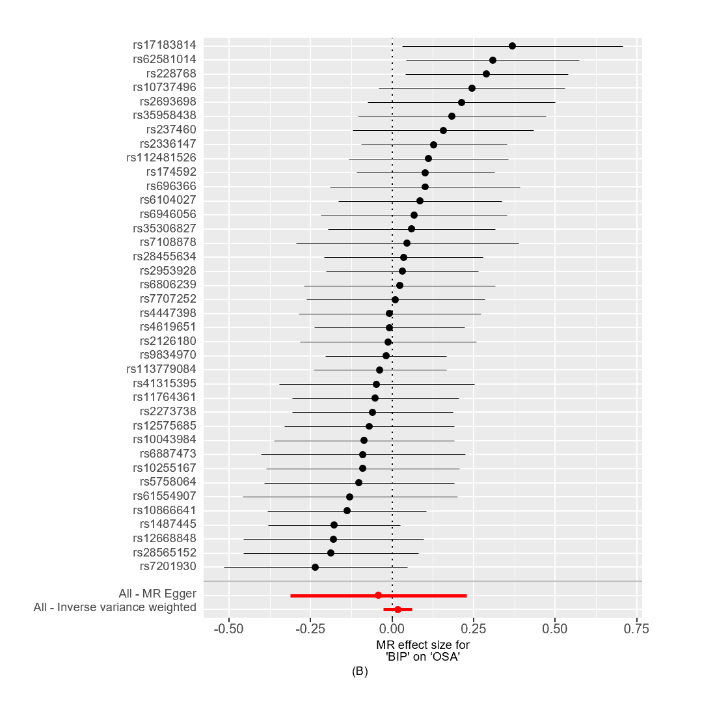
3. Forest plots for psychiatric disorders on obstructive sleep apnea.


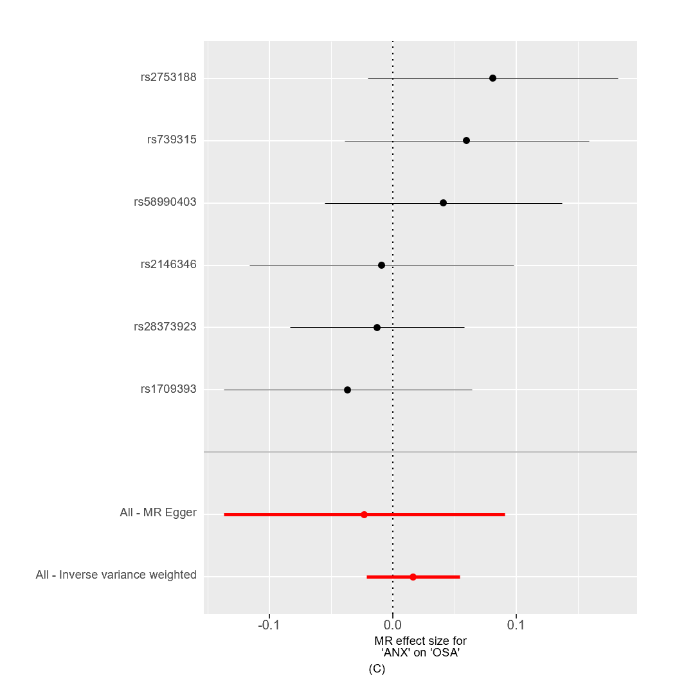

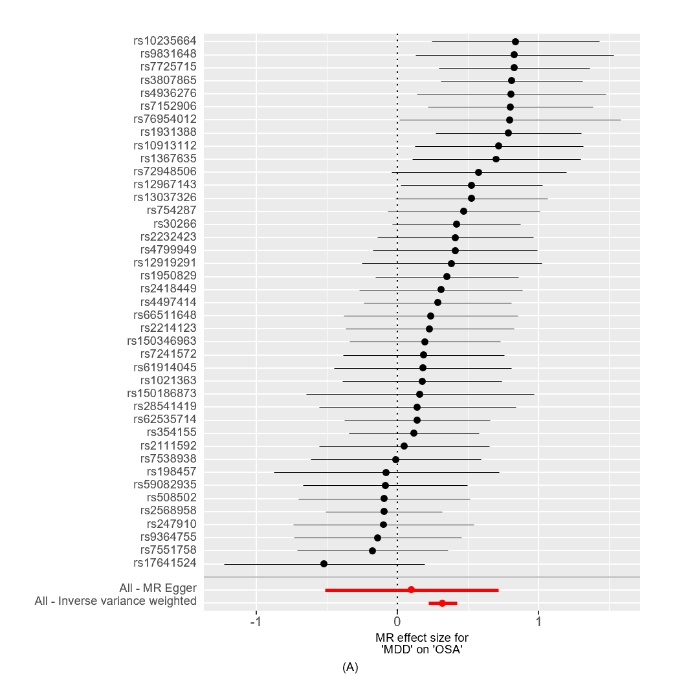


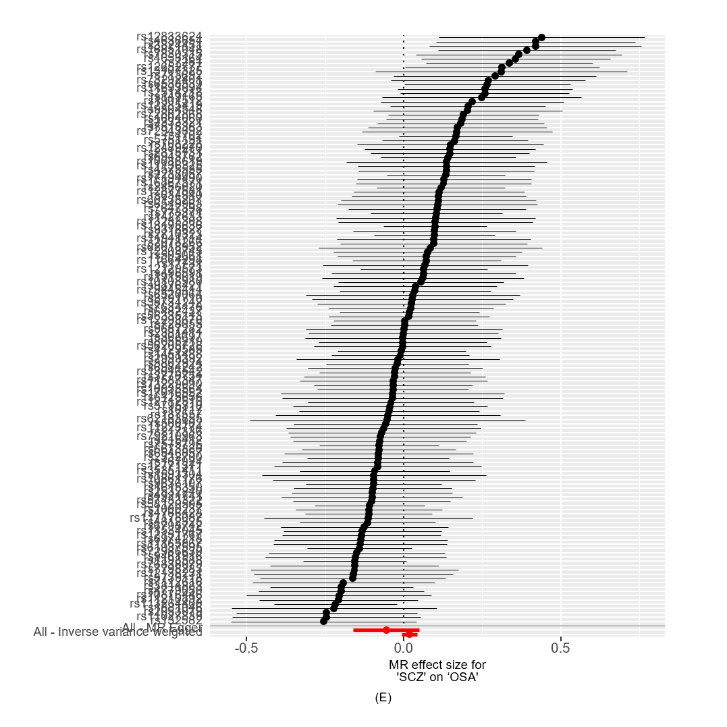


Abbreviations: MDD, major depressive disorder; SCZ, schizophrenia; BIP, bipolar disorder; ANX, anxiety disorder; PTSD, post-traumatic stress disorder; OSA, obstructive sleep apnea.
