# Supplementary material for: Assessing the causal relationship between psychiatric disorders and obstructive sleep apnea: a bidirectional Mendelian randomization
Source: Front Psychiatry. 2024 Feb 14;15:1351216. doi: 10.3389/fpsyt.2024.1351216 (PMC10903261; doi:10.3389/fpsyt.2024.1351216)
Supplement: Supplementary file 2 [file DataSheet_2.docx]

**Supplementary Figure S2**


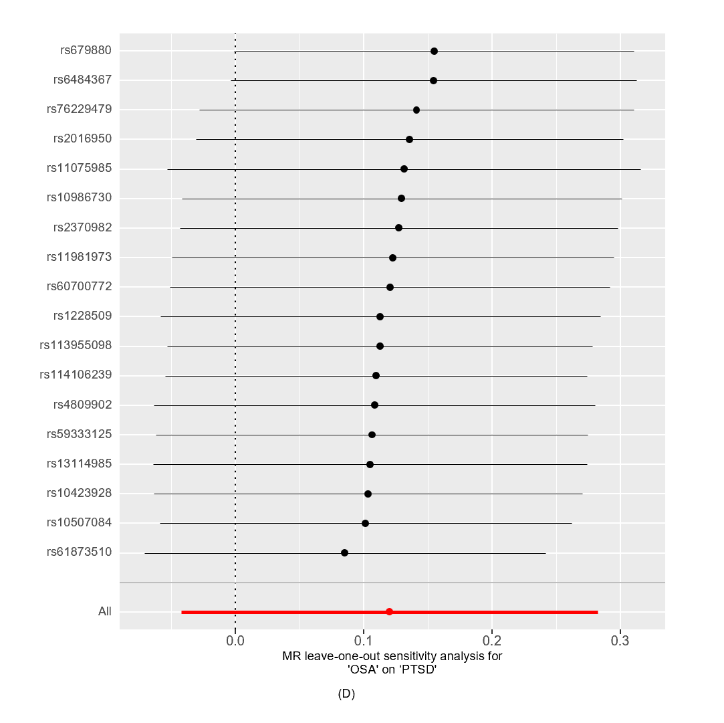

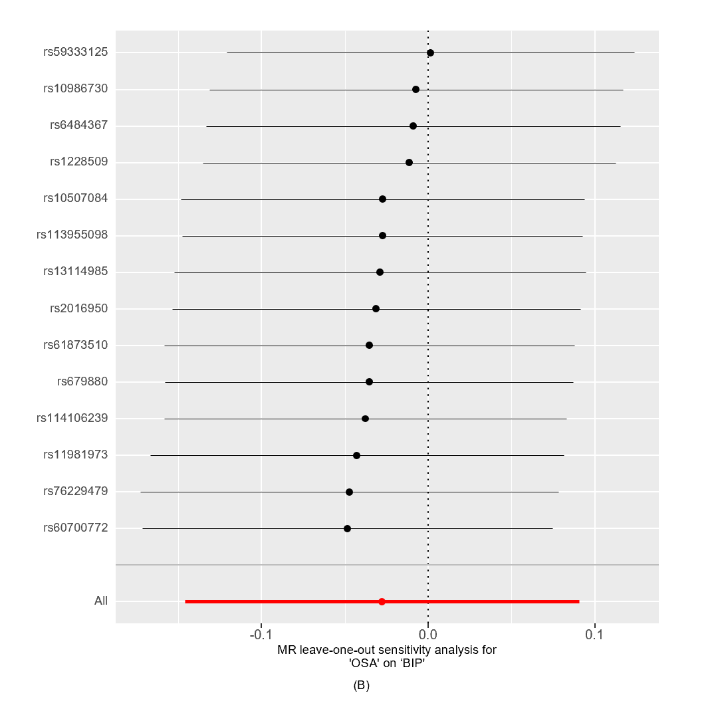
1.Leave-one-out analysis for obstructive sleep apnea on psychiatric disorders.


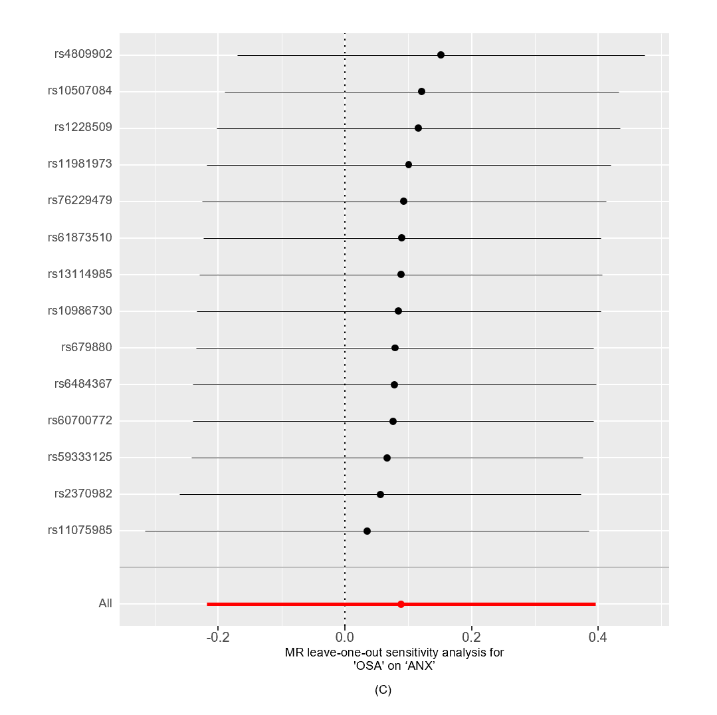

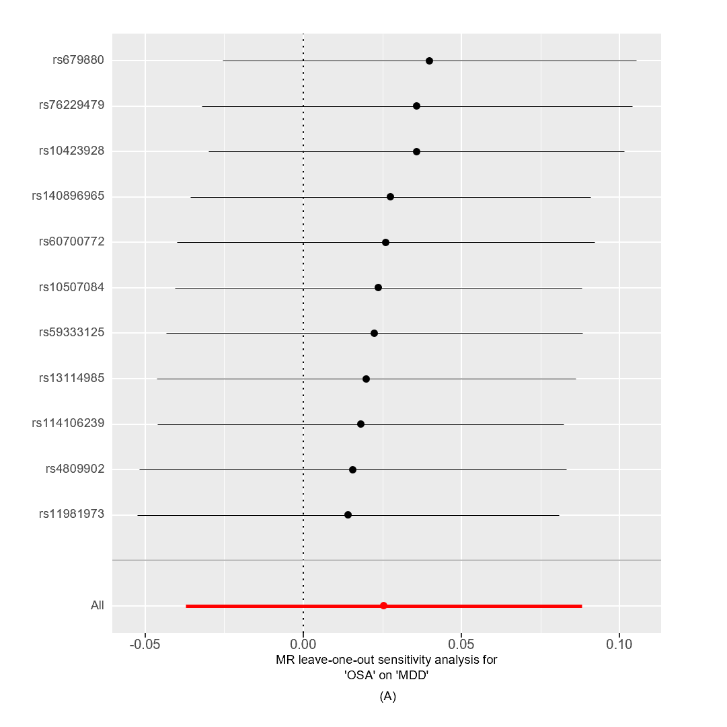


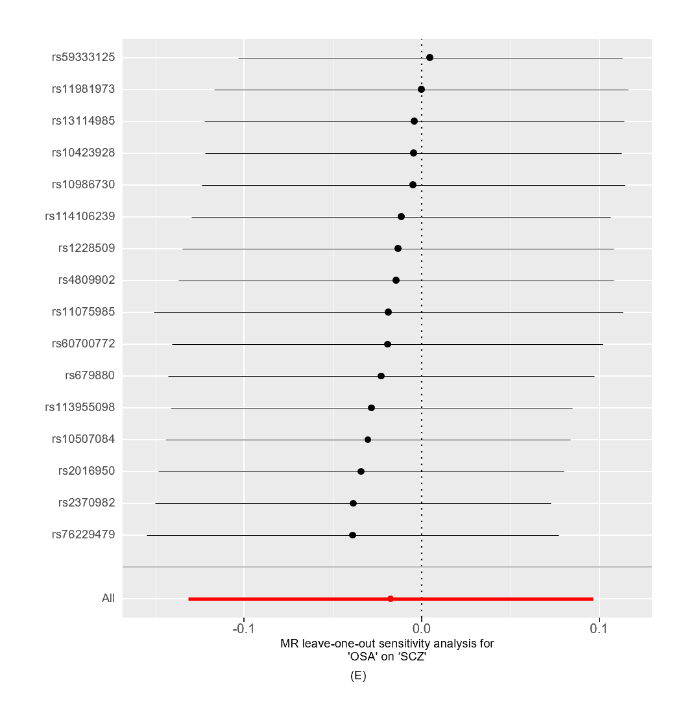


Abbreviations: MDD, major depressive disorder; SCZ, schizophrenia; BIP, bipolar disorder; ANX, anxiety disorder; PTSD, post-traumatic stress disorder; OSA, obstructive sleep apnea.


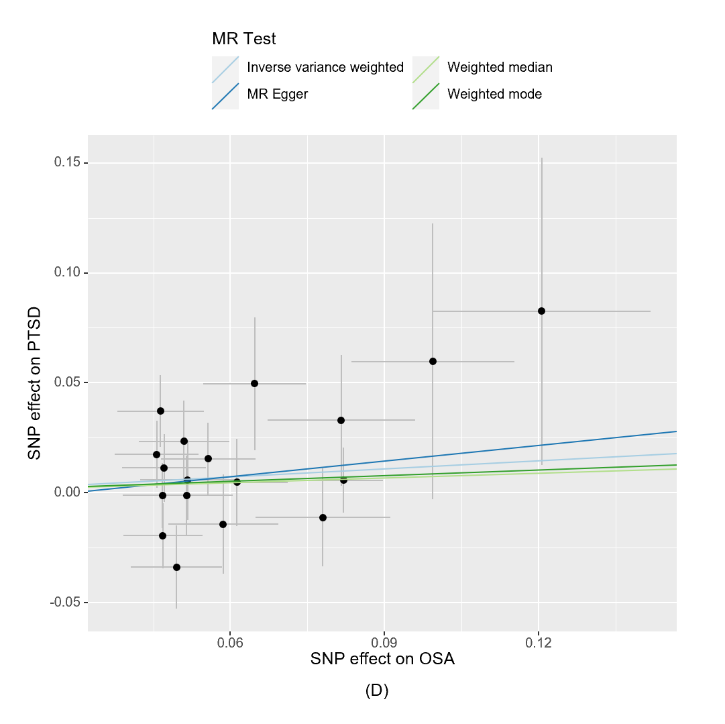

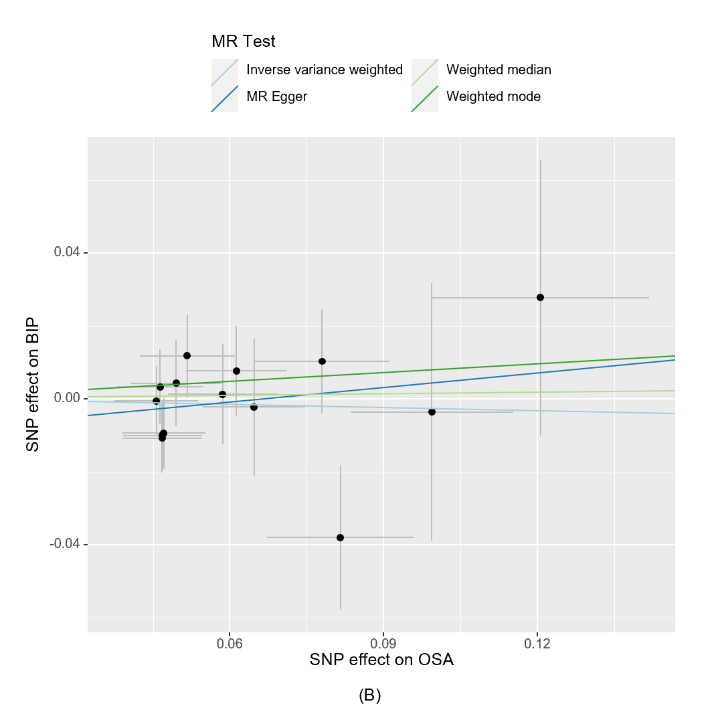
2. Scatter plots for obstructive sleep apnea on psychiatric disorders.


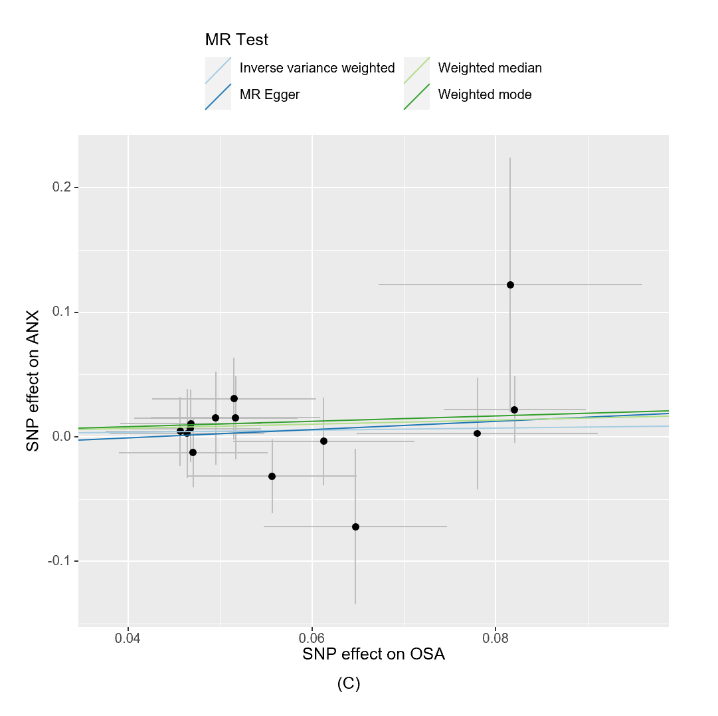

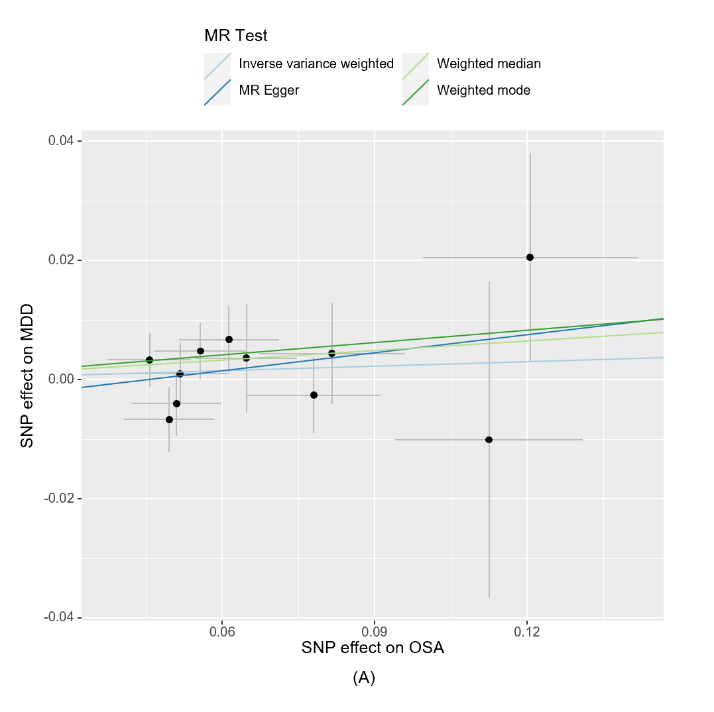


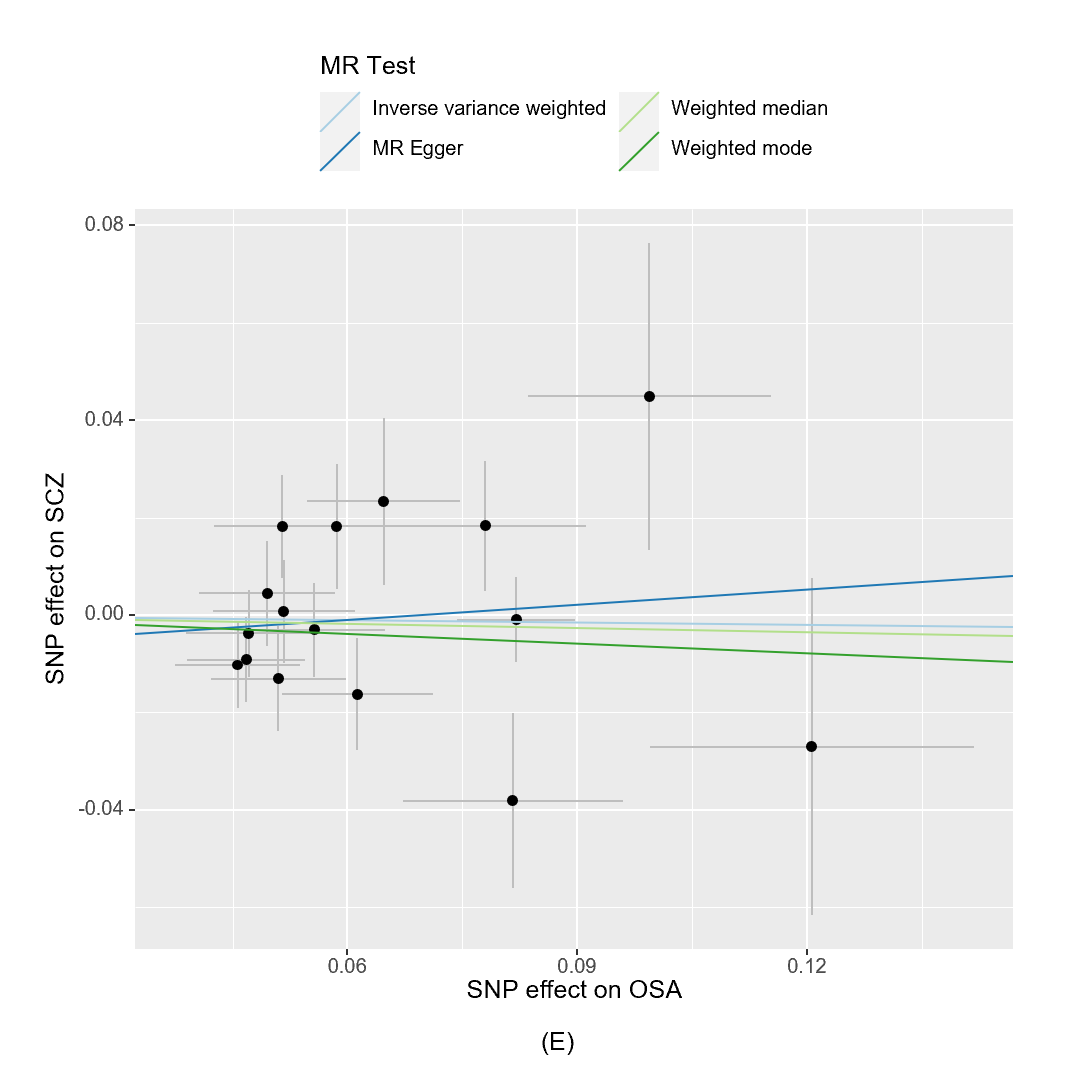


Abbreviations: MDD, major depressive disorder; SCZ, schizophrenia; BIP, bipolar disorder; ANX, anxiety disorder; PTSD, post-traumatic stress disorder; OSA, obstructive sleep apnea.


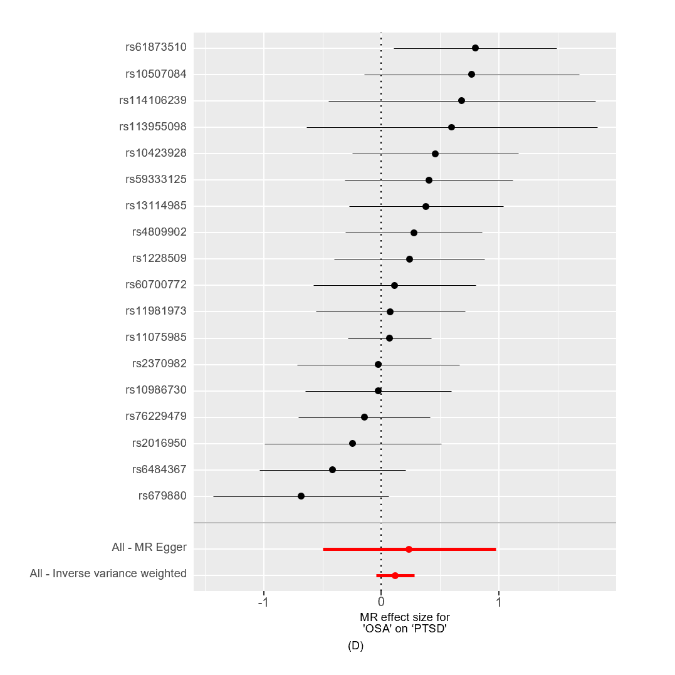

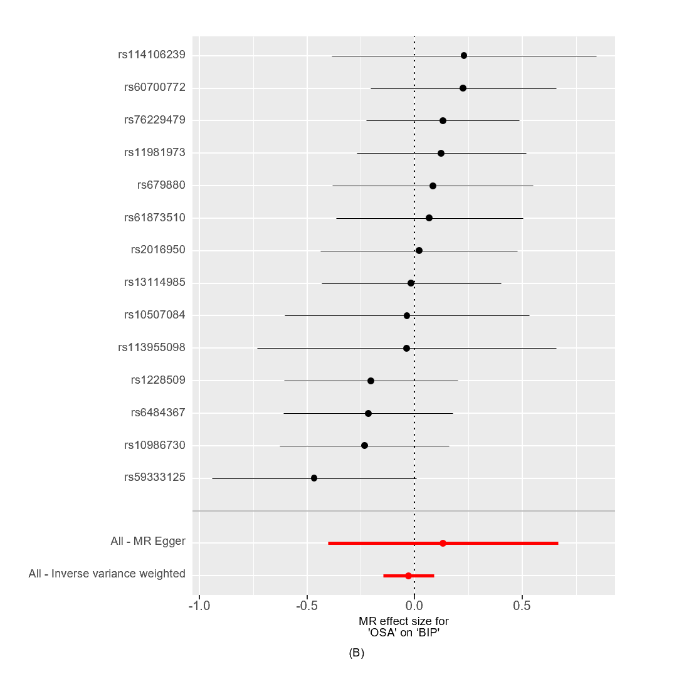
3. Forest plots for obstructive sleep apnea on psychiatric disorders.


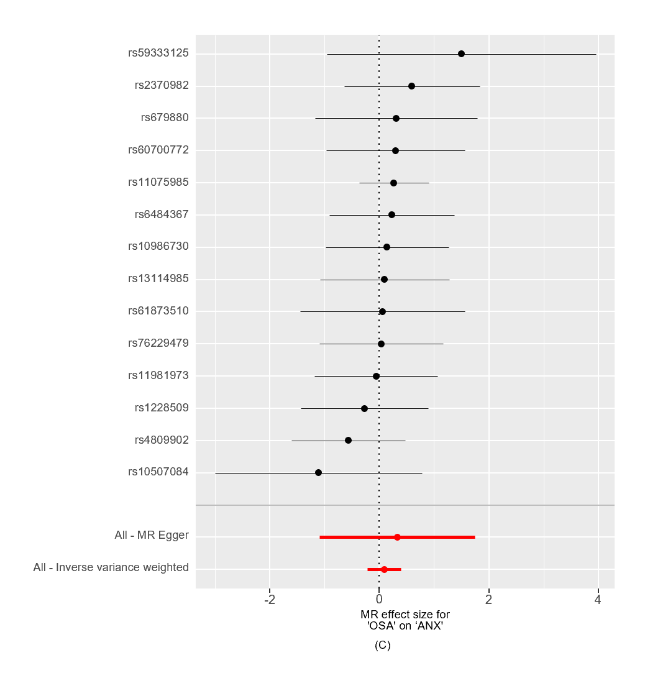

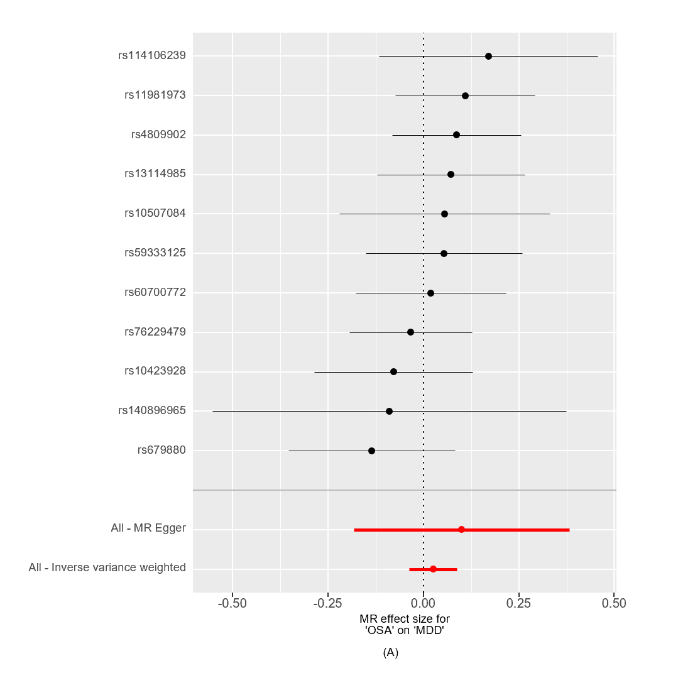


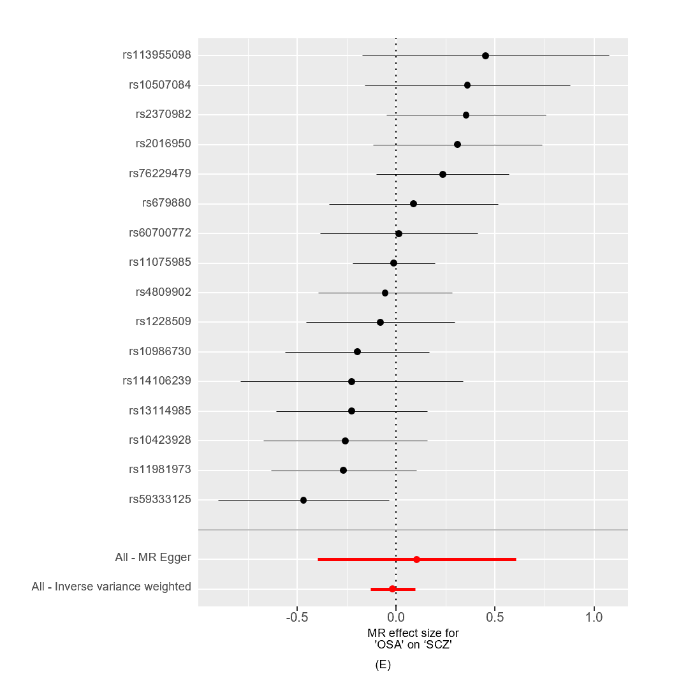


Abbreviations: MDD, major depressive disorder; SCZ, schizophrenia; BIP, bipolar disorder; ANX, anxiety disorder; PTSD, post-traumatic stress disorder; OSA, obstructive sleep apnea.
